# Context-dependent amygdala–prefrontal connectivity during the dot-probe task varies by irritability and attention bias to angry faces

**DOI:** 10.1038/s41386-022-01307-3

**Published:** 2022-06-01

**Authors:** Reut Naim, Simone P. Haller, Julia O. Linke, Allison Jaffe, Joel Stoddard, Matt Jones, Anita Harrewijn, Katharina Kircanski, Yair Bar-Haim, Melissa A. Brotman

**Affiliations:** 1 Neuroscience and Novel Therapeutics Unit, Emotion and Development Branch, National Institute of Mental Health, Bethesda, MD USA; 2 Section on Mood Dysregulation and Neuroscience, Emotion and Development Branch, National Institute of Mental Health, Bethesda, MD USA; 3 Pediatric Mental Health Institute, Children’s Hospital Colorado, Department of Psychiatry & Neuroscience Program, University of Colorado, Anschutz Medical Campus, Aurora, CO USA; 4 Department of Psychology and Neuroscience, University of Colorado at Boulder, Boulder, CO USA; 5 Section on Development and Affective Neuroscience, Emotion and Development Branch, National Institute of Mental Health, Bethesda, MD USA; 6 School of Psychological Sciences, Tel-Aviv University, Tel-Aviv, Israel

**Keywords:** Human behaviour, Attention, Amygdala

## Abstract

Irritability, defined as proneness to anger, is among the most common reasons youth are seen for psychiatric care. Youth with irritability demonstrate aberrant processing of anger-related stimuli; however, the neural mechanisms remain unknown. We applied a drift-diffusion model (DDM), a computational tool, to derive a latent behavioral metric of attentional bias to angry faces in youth with varying levels of irritability during functional magnetic resonance imaging (fMRI). We examined associations among irritability, task behavior using a DDM-based index for preferential allocation of attention to angry faces (i.e., extra-decisional time bias; Δ*t*_0_), and amygdala context-dependent connectivity during the dot-probe task. Our transdiagnostic sample, enriched for irritability, included 351 youth (ages 8–18; *M* = 12.92 years, 51% male, with primary diagnoses of either attention deficit/hyperactivity disorder [ADHD], disruptive mood dysregulation disorder [DMDD], an anxiety disorder, or healthy controls). Models accounted for age, sex, in-scanner motion, and co-occurring symptoms of anxiety. Youth and parents rated youth’s irritability using the Affective Reactivity Index. An fMRI dot-probe task was used to assess attention orienting to angry faces. In the angry-incongruent vs. angry-congruent contrast, amygdala connectivity with the bilateral inferior frontal gyrus (IFG), insula, caudate, and thalamus/pulvinar was modulated by irritability level and attention bias to angry faces, Δ*t*_0_, all *t*s_350_ > 4.46, *p*s < 0.001. In youth with high irritability, elevated Δ*t*_0_ was associated with a weaker amygdala connectivity. In contrast, in youth with low irritability, elevated Δ*t*_0_ was associated with stronger connectivity in those regions. No main effect emerged for irritability. As irritability is associated with reactive aggression, these results suggest a potential neural regulatory deficit in irritable youth who have elevated attention bias to angry cues.

## Introduction

Irritability, characterized by increased proneness to anger, is among the most common reasons that families seek psychiatric care [1, 2]. Irritability is highly impairing and common, implicated in multiple clinical conditions in youth, and is associated with negative outcomes in adulthood including high suicidality, depression and anxiety disorders, low income, and poor academic performance [3, 4]. However, the cognitive and neural underpinnings of irritability remain largely unknown [1, 2, 5, 6]. Using clinically-relevant stimuli (e.g., angry faces), research shows that youth with irritability and elevated trait anger demonstrate cognitive biases towards anger-related cues [7–10]. Likewise, a low threshold for negative cue detection has been associated with reactive aggression and temper outbursts [11], characteristics of irritable youth. Here, we applied a Drift Diffusion Model (DDM), a class of computational modeling to identify associations among irritability, anger-related attention bias, and neural connectivity during angry-face processing.

Attention orienting, the ability to detect and modulate responses to salient negative environmental cues, is mediated by amygdala-prefrontal circuitry [6, 12–15] and is impaired in youth with psychopathology [16, 17]. Irritable youth show an attention bias toward angry faces [7, 9, 10], which has been associated with emotional dysregulation and aggressive behaviors [7]. The dot-probe task is a canonical paradigm that assesses selective attention and attention biases [16, 18]; the participant responds to a probe that appears in a location previously occupied by an emotionally-relevant (congruent) or neutral (incongruent) cue. Attentional biases have been typically operationalized as the difference between mean reaction time to probes appearing in emotional (congruent) versus neutral (incongruent) stimulus locations [18, 19]. However, this approach provides a general, coarse metric of bias that shows low retest reliability [20, 21] and does not model key performance-relevant data.

Computational modeling has been harnessed to parse more nuanced cognitive parameters associated with attentional processes [22–24]. These approaches account for the full distribution of behavioral reaction times and response choices, and can be used to compute discrete parameters reflecting different cognitive components associated with task performance. Recent work shows that an attention bias index derived from the extra-decisional time component of the DDM yields improved test-retest and split-half reliabilities relative to the aforementioned classic attention bias score [22], suggesting that this component may provide a more reliable metric of attention deployment to emotionally-relevant stimuli.

Using a DDM to compute an attention bias index, this fMRI study examines the neurobiology mediating attentional deployment in the angry-incongruent vs. angry-congruent contrast for youth with varying levels of irritability. Neural circuitry involving the amygdala and the prefrontal cortex has been associated with attention allocation [14, 15, 17, 25–27] and with the processing and the regulation of negative emotions [28–32] and anger [33–35]. Specifically for youth with psychopathology, decreased amygdala-medial prefrontal cortex connectivity has been found in irritability in the context of negative cues [12]. Additionally, a translational model of irritability [11] posits that amygdala-frontal dysfunction mediates irritability. Based on this theoretical framework and preliminary findings, we anticipated aberrancies in this circuitry in irritable youth during attentional processing of angry-face stimuli. Additionally, the inferior frontal gyrus (IFG) is a region with a critical role in the allocation and shifting of attentional resources [36–38]. Thus, we predicted that highly irritable youth, characterized by difficulty in regulating anger, will present decreased amygdala-prefrontal connectivity, and specifically amygdala-IFG connectivity in the angry-incongruent vs. angry-congruent contrast.

This study comprises a large transdiagnostic sample of pediatric patients and healthy controls, capturing a wide spectrum of irritability. First, we leveraged computational modeling to identify the precise component of anger-related attention bias, using the extra-decisional time parameter of the DDM. Second, we examined whether this behavioral metric is associated with amygdala seed-based connectivity and tested whether this association varies with level of irritability. We focused on context-dependent amygdala functional connectivity, performing a generalized psychophysiological interaction (gPPI) [39–41]. Identifying the neural circuitry associated with anger-related attentional processing in irritability may advance neurocognitive, mechanism-based interventions.

As irritability and anxiety symptoms commonly co-occur [42] and research demonstrates aberrant attentional processing in anxiety [15, 22, 43], it is essential to account for anxiety when exploring irritability. Therefore, anxiety was accounted for in statistical analyses.

## Materials and methods

### Participants

A total of 464 youth enrolled. *N* = 113 were excluded primarily due to poor behavioral or neural data (eMethods 1). The final sample consisted of 351 youth (Age: 8.00–18.00 years; *M* = 12.92; standard deviation [SD] = 2.66). Of these 351, data from *N* = 190 (54.13%) were previously published in a study examining overlapping vs. unique neural correlates of irritability and anxiety, using a bifactor analytic approach to parse clinical phenomena [6]. The present study, which almost doubles the participants included in Kircanski and colleagues [6], applies an advanced computational approach to behavioral performance to probe aberrant attentional processing in irritability. Specifically, the sample includes a larger number of patients with ADHD and DMDD, psychopathologies particularly associated with irritability (see Table 1). Data from *N* = 161 (*N* = 96 patients) have not been examined. Parents/children gave written informed consent/assent. Participants received monetary compensation. The NIMH IRB approved all study procedures. Data were acquired between June 30, 2012, and November 30, 2019.

**Table 1:** Demographics and clinical characteristics.

Characteristic (*N* = 351)	
Sex, % Male	51.00%
Age (years)	
Mean (SD)	12.92 (2.66)
Range	8.00−18.00
Race, *N* (%)	
White	232 (66.09)
Multiracial	45 (12.82)
African American	43 (12.25)
Unknown	20 (5.69)
Asian	8 (2.27)
Native American or Alaskan Native	2 (0.56)
Native Hawaiian and Other Pacific Islanders	1 (0.28)
Ethnicity, *N* (%)	
Not Hispanic or Latino	301 (85.75)
Hispanic or Latino	39 (11.11)
Unknown	11 (3.13)
SES	
Mean (SD)	35.61 (18.93)
Range	20.00−120.00
IQ	
Mean (SD)	112.46 (12.95)
Range	70−143
Irritability Parent-Child Mean (ARI)	
Mean (SD)	2.83 (2.72)
Range	0.00–12.00
Irritability Child Rated (ARI)	
Mean (SD)	2.53 (2.80)
Range	0.00–12.00
Irritability Parent Rated (ARI)	
Mean (SD)	3.14 (3.36)
Range	0.00–12.00
Anxiety Parent-Child Mean (SCARED)	
Mean (SD)	17.17 (13.21)
Range	0.00–56.50
Anxiety Child Rated (SCARED)	
Mean (SD)	17.93 (14.89)
Range	0.00–69.00
Anxiety Parent Rated (SCARED)	
Mean (SD)	16.42 (14.66)
Range	0.00–75.00
Medication, N (%)	
None	250 (71.12)
Stimulants	74 (21.10)
SSRI (Anti-Depressant)	30 (8.54)
SGA (Anti-Psychotic)	14 (3.98)
AED	14 (3.98)
No Information	14 (3.98)
Primary Diagnosis, N (%)	
Anxiety Disorder	115 (32.76)
Healthy Volunteer	109 (31.10)
DMDD	65 (18.51)
ADHD	62 (17.77)
Average motion after censoring	
Mean (SD)	0.035 (0.04)
Range	0.00–0.19

	Male	Female
Genital/Breast development		
Mean (SD)	3.08 (1.45)	3.18 (1.43)
Range	1.00–5.00	1.00–5.00
Pubic hair development		
Mean (SD)	2.94 (1.60)	3.35 (1.60)
Range	1.00–5.00	1.00–5.00

Data were missing for *N* = 39 on the SES; for *N* = 15 on the WASI; for *N* = 5 on the Child-reported ARI; for *N* = 2 on the Parent-reported ARI; for *N* = 3 on the Child-reported SCARED; for *N* = 2 on the Parent-reported SCARED; and for *N* = 9 on the medication report. Participants could be medicated with more than one type of medication.

Data were missing for *N* = 114. *ADHD* attention deficit hyperactivity disorder, *AED* antiepileptic drugs, *ARI* Affective Reactivity Index, *DMDD* disruptive mood dysregulation disorder, *SCA*.*RED* screen for child anxiety related emotional disorders, *SES* socioeconomic status, SGE second generation antipsychotics, *SSRI* selective serotonin reuptake inhibitors. Pubertal status is based on the Tanner scale.

### Clinical characteristics

Patients were diagnosed with a primary anxiety disorder (generalized, social, or separation anxiety disorder; *N* = 115), attention-deficit/hyperactivity disorder (ADHD; *N* = 62), or disruptive mood dysregulation disorder (DMDD; N = 65), diagnoses associated with irritability [44, 45]. Sample included 109 healthy controls to maximize variability in irritability, the clinical dimension of interest. For detailed inclusion/exclusion criteria see eMethods 1. Table 1 summarizes participant characteristics and provides information on pubertal status based on the Tanner scale [46]. Most participants were in early- to mid- pubertal stages, with no significant difference between females and males (*t*_(231)_ = 1.28, *p*  =  0.20).

### Symptom measures

Irritability was assessed using the Affective Reactivity Index (ARI) [47] and anxiety using the Screen for Child Anxiety Related Emotional Disorders (SCARED) [48] (eMethods 2). Averaged scores between youth- and parent-reported ARI and SCARED were generated [12, 49]. There were positive correlations between youth- and parent-reported irritability (*r*_351_ = 0.56, *p* < 0.001) and between average scores of youth- and parent-reports on irritability and anxiety (*r*_351_ = 0.46, *p* < 0.001). The Conners’ Parent Rating Scale [50] was used for post-hoc analyses covarying for ADHD (eResults 3).

### Dot-Probe task

**Fig. 1:**
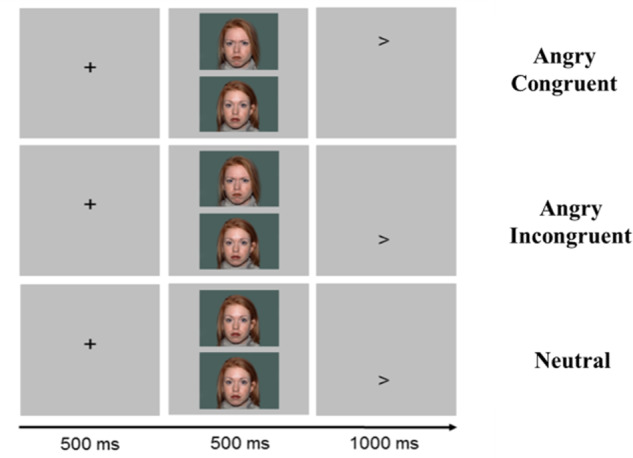
Dot-probe task overview. Dot-Probe Task Schematic. A pair of faces (either angry-neutral or neutral-neutral) is presented on each trial, followed by a probe (< or >). Participants indicate the probe direction using a button press. The task was provided by the Tel-Aviv University/NIMH Attention Bias Modification Treatment Initiative (http://people.socsci.tau.ac.il/mu/anxietytrauma/research/).

Participants performed a dot-probe task adapted from the Tel-Aviv University/NIMH ABMT Initiative (http://people.socsci.tau.ac.il/mu/anxietytrauma/research/) during fMRI (Fig. 1). Task includes three trial types: (1) angry-congruent; (2) angry-incongruent; (3) neutral-neutral (eMethods 1).

### Drift Diffusion Model

A DDM was applied to quantify distinct components of task performance (eMethods 1) [51]. Previous studies applying DDM to the dot-probe task have focused on estimating the drift rate parameter (*v*), reflecting efficiency of information processing, and the extra-decisional time parameter (*t*_0_), reflecting a nondecision component that includes perception duration, stimulus encoding, and motor execution [51, 52]. *t*_0_ is particularly relevant for the measurement of attention processing [22] because the affective cues are presented prior to the probe and the initiation of the target-dependent decision process. The difference in *t*_0_ between incongruent and congruent trials (Δ*t*_0_) includes the time during incongruent trials to orient attention away from the angry face display to the target probe in the opposite location, compared to the congruent condition. The angry-incongruent reflects a condition where the probe is in a different place than where the angry face was previously presented. Therefore, Δ*t*_0_ represents the influence of the anger-related stimulus on attention orienting [22, 52, 53].

Models were fit to the data using fast-dm software version 30.2 [54]. Δ*t*_0_ scores were calculated by subtracting *t*_0_ parameters in the congruent condition from *t*_0_ parameters in the incongruent condition (*t*_0 incongruent_ − *t*_0 congruent_) [22]. Larger scores indicate greater anger-related attention bias.

Behavioral analyses included a Spearman correlation and t-tests to assess associations between irritability and age or sex, respectively. To measure associations between anger-related attentional bias and irritability, controlling for covariates, a multivariate linear regression model was applied, including irritability, anxiety, age, and sex as predictors. Δ*t*_0_ was the outcome variable. To assess associations between irritability and the *t*_0_ parameter across specific task conditions (congruent/incongruent/neutral), general linear models (GLMs) were used to model *t*_0_ for each task condition as a function of irritability, covarying for anxiety and age.

### Neuroimaging acquisition, preprocessing, and analysis

fMRI data were acquired on General Electric 3-T MR750 imaging systems (Waukesha, Wisconsin, USA) with either an 8- or 32-channel head coil. Functional images were analyzed using Analysis of Functional NeuroImages (AFNI) [55]. See Supplement for details on acquisition and first-level analyses (eMethods 3). AFNI’s 3dMVM [56] was used for group-level analyses. GLMs estimating BOLD response and gPPI were used to assess voxel-wise functional connectivity of each left and right amygdala seed as a function of task condition [40, 41]. Specifically, gPPI was used to estimate the magnitude of the seed-time series of amygdala connectivity in the context of the task conditions. Since the angry-incongruent vs. angry-congruent was the a-priori contrast of interest, gPPI was restricted to this contrast, calculated as incongruent minus congruent. Models included Δ*t*_0_, irritability, and the interaction between Δ*t*_0_ and irritability (grand-mean centered) as between-subject independent variables. Age, average in-scanner motion, and anxiety score (all grand-mean centered), as well as sex and head-coil during acquisition (dummy coded) were covariates.

Results were thresholded voxel-wise at *p* < 0.005. Cluster correction was used to control for multiple tests via 3dClustSim (nearest neighbor = 1) and was set to *α* = 0.05 for activation and to *α* = 0.025 for functional connectivity (Bonferroni corrected for 2 seeds). We used AFNI’s 3dFWHMx with -acf flag to estimate the smoothness of the residuals via Monte Carlo cluster-size simulation with a Gaussian plus mono-exponential spatial autocorrelation function. Parameters were estimated and averaged for all participants, yielding an effective smoothness of FWHM = 9.14 mm (ACF parameters, *a* = 0.58, *b* = 3.42, and *c* = 10.81), resulting in a cluster-size threshold of *k* > 60 (1078 mm^3^) for activation and *k* > 71 (1313 mm^3^) for connectivity.

For post-hoc analyses, mean activity and connectivity values for significant clusters of the incongruent vs. congruent contrast were extracted using AFNI’s 3dROIstat program. Using SPSS (version 23.0; SPSS Inc) [57], multivariate linear regression models were conducted using the same variables as the fMRI group analyses. fMRI analyses were then replicated adding medication usage (dummy-coded) as a covariate to the model to test for potential influence of medication status on the results.

Three sets of additional analyses were conducted (eResults 1–3, eTable 2–3). First, fMRI analyses were replicated adding an anxiety-by-irritability interaction as an additional term; these yielded similar results to the initial models, which included anxiety as a covariate. Second, analyses examined associations of brain function with irritability and Δ*t*_0_, covarying for ADHD symptoms. All initial findings survived this addition. Third, behavioral and fMRI analyses with the traditional anger-related attention-bias scores were performed.

## Results

### Demographics and behavior

Age was negatively associated with irritability (*r*_s351_ = −0.15, *p* = 0.005). Sex was significantly associated with irritability (*t*_349_ = −2.50_,_ *p* = 0.013); males exhibited higher irritability levels compared to females. No associations were found between irritability and race, ethnicity, IQ, socioeconomic status (SES) (all *p*s > 0.554), or medication status (η^2^ = 0.240). Adjusting for covariates, no significant findings emerged for the association between irritability and Δ*t*_0_ (*p* = 0.890). Using a categorical approach to compare Δ*t*_0_ among diagnostic groups, no significant differences were found (*p* = 0.240). Additionally, *t*_0_ across task conditions was not significantly related to irritability (*p*s > 0.244). See eTable 1 for descriptive statistics of DDM parameters.

Spearman-Brown split-half reliability of Δ*t*_0_ in the current sample was 0.20; for the conventional bias score it was 0.02. Fisher’s *r*-to-*z* test comparing these reliability scores was significant (z = 2.41, *p* = 0.016), suggesting that though the reliability of Δ*t*_0_ is low, it may nonetheless be a somewhat improved index compared to the conventional bias.

### Amygdala seed-based gPPI functional connectivity

**Fig. 2:**
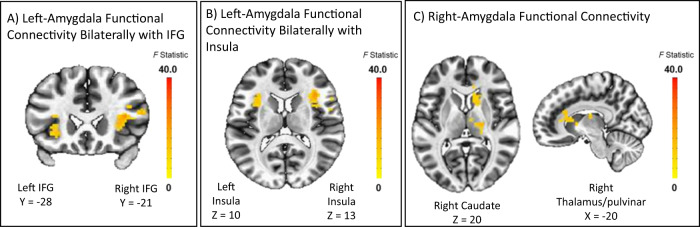
Amygdala functional connectivity during orienting to angry faces as a function of extra-decisional time bias (Δt_0_) by irritability. Functional left amygdala connectivity bilaterally to the IFG during orienting to angry stimuli is shown in A. Functional left amygdala connectivity bilaterally to the insula during orienting to angry stimuli is shown in B. Functional right amygdala connectivity to the right caudate and the right thalamus/pulvinar during orienting to angry face stimuli is shown in C; Note: Z, Y, X indicate the peak location. Note: these are not individual data; informed consent for publishing the data was obtained from the participants.

**Fig. 3:**
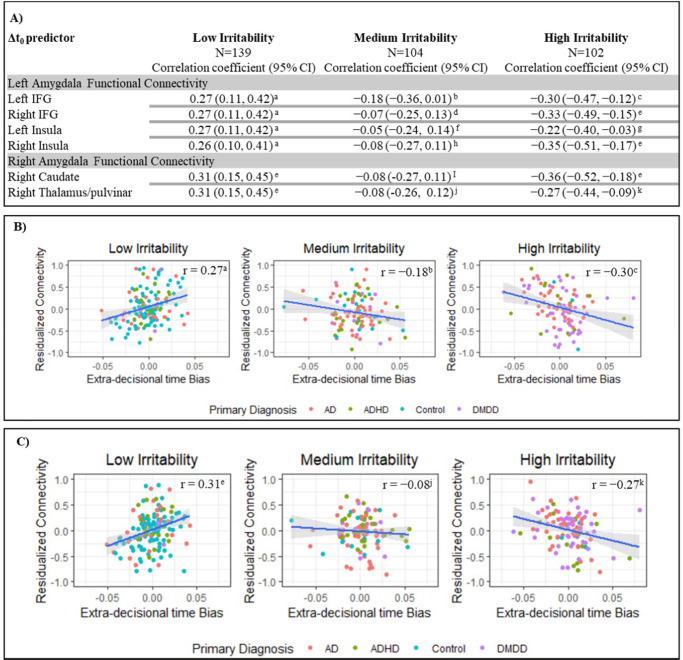
Correlations between extra-decisional time bias (Δ*t*_0_) and amygdala functional connectivity as a function of irritability. **A** It shows the correlation coefficients for the interaction between extra-decisional time bias (Δ*t*_0_) and residualized functional connectivity for the left amygdala bilaterally with the IFG and insula; and the right amygdala with the right caudate and the right thalamus/pulvinar, for varying levels of irritability. **B** It illustrates the data for the correlation coefficients listed in table A for left amygdala functional connectivity with the left IFG during orienting to angry faces. **C** This illustrates the data for the correlation coefficients listed in Table A for right amygdala functional connectivity with the right thalamus/pulvinar during orienting to angry faces. The effects of motion, age, sex, head-coil channel during acquisition, and SCARED scores were partialled out from mean change in connectivity. Resultant residual change in connectivity during orienting to angry face stimuli by Δ*t*_0_ for individuals grouped into tertiles of ARI scores (averaged across youth and parent report). Correlation coefficients are given for the plotted data. Primary diagnosis abbreviations are as follows: Anxiety Diagnosis (AD), Attention Deficit Hyperactivity Disorder (ADHD), Healthy Volunteers (Control), Disruptive Mood Dysregulation disorder (DMDD). a *P* = 0.001. b *P* = 0.06. c *P* = 0.002. d *P* = 0.50. e *P* < 0.001. f *P* = 0.60. g *P* = 0.02. h *P* = 0.40. i *P* = 0.39. j *P* = 0.43. k *P* = 0.005.

Greater Δ*t*_0_ was associated with increased left amygdala functional connectivity with bilateral IFG and insula in the angry-incongruent vs. angry-congruent contrast (*βs*_350_ > 4.71, *ts*_350_ > 2.78, *ps* = 0.001). However, this main effect was qualified by significant higher-order interactions. Significant interactions were observed between Δ*t*_0_ and irritability for left amygdala functional connectivity with bilateral IFG and insula (Fig. 2A, B; right IFG: *β*_350_ = −1.84, *t*_350_ = −4.74, *p* < 0.001; left IFG: *β*_350_ = −1.89, *t*_350_  = −4.69, *p* < 0.001; right insula: *β*_350_ = −1.92, *t*_350_  = −5.53, *p* < 0.001; left insula: *β*_350_ = −1.71, *t*_350_ = −4.47, *p* < 0.001). As irritability increased, the association between Δ*t*_0_ and amygdala functional connectivity with these regions became more negative. To further explore these interactions, the sample was grouped into tertiles of low (ARI score cutoff: 1.5), medium (ARI score cutoff: 4.5) and high irritability (ARI score cutoff: above 4.5). For youth with relatively elevated irritability, greater Δ*t*_0_ was associated with a weaker left amygdala connectivity to these regions, whereas for youth with relatively low irritability, greater Δ*t*_0_ was associated with stronger connectivity (Table 2, Fig. 3A, B).

**Table 2:** Brain regions showing significant main and interaction effects.

		Peak MNI Coordinates (Icent)						
Angry Incongruent vs. Congruent	*K*	*X*	*Y*	*Z*	Size, mm^3^	*t* Statistic	*P* value	Location
Whole-Brain BOLD Activity								
Δ*t*_0_	247	−25	67	23	3858	−4.61	<0.001	Right Precuneus
Right Amygdala Seed Functional Connectivity								
Δ*t*_0_ by Irritability PC mean	122	−10	−26	20	1906	−5.30	<0.001	Right Caudate
	99	−20	29	10	1546	−5.06	<0.001	Right Thalamus/pulvinar
Left Amygdala Seed Functional Connectivity								
Δ*t*_0_ by Irritability PC mean	190	−37	−23	13	2968	−5.53	<0.001	Right (anterior) Insula
	103	−52	−21	18	1609	−4.74	<0.001	Right IFG
	92	37	−28	3	1437	−4.69	<0.001	Left IFG
	81	37	−11	10	1265	−4.47	<0.001	Left (anterior) Insula

*p* value represents significance value from post hoc multivariate linear regression on mean BOLD signal for extracted cluster. Location represents anatomical overlap of cluster with region.

Δ*t*_0_ Extra-decisonal time bias, *BOLD* blood oxygenation-level dependent, *PC* parent child.

Greater Δ*t*_0_ was also associated with increased right amygdala functional connectivity with the right caudate and right thalamus/pulvinar in the angry-incongruent vs. angry-congruent contrast (*βs*_350_ > 5.27, *ts*_350_ > 3.67, *ps* < 0.001). Again, this was qualified by significant interactions between Δ*t*_0_ and irritability for right amygdala connectivity with right caudate and right thalamus/pulvinar (Table 2, Fig. 2C; right caudate: *β*_350_ = −1.81, *t*_350_ = −5.30, *p* < 0.001; right thalamus/pulvinar: *β*_350_ = −1.70, *t*_350_ = −5.06, *p* < 0.001). In youth with relatively elevated irritability, greater Δ*t*_0_ was associated with a weaker right amygdala connectivity to these regions, whereas for youth with relatively low irritability, greater Δ*t*_0_ was associated with stronger connectivity (Fig. 3A, 4c). No other significant findings for amygdala connectivity emerged.

Post-hoc analyses adding medication usage as a covariate revealed similar results to the initial model of significant interactions between Δ*t*_0_ and irritability for amygdala connectivity (*βs*_350_ < −1.70, *ts*_350_ < −4.43, *ps* < 0.001).

### Activation

Whole-brain analysis revealed that greater Δ*t*_0_ scores were negatively associated with activation in the right precuneus (*β*_350_ = −0.71, *t*_350_ = −4.61, *p* < 0.001) in the angry-incongruent vs. angry-congruent contrast (eFigure 3). No findings emerged for individual differences in irritability. Table 2 presents brain regions showing a significant main effect of Δ*t*_0_. Post-hoc analyses adding medication usage as a covariate revealed a similar result (*β*_350_ = −0.70, *t*_350_ = −4.09, *p* < 0.001).

## Discussion

The current study applied computational modeling to examine neural function during the dot-probe task in a large sample of youth with varying levels of irritability. Leveraging DDMs to parse behavior, we discovered a complex three-way interaction between context-dependent amygdala functional connectivity, irritability, and attention bias to angry faces. As irritability increased, the relation between attention bias (i.e., Δ*t*_0_) and amygdala functional connectivity in the angry-incongruent vs. angry-congruent contrast became more negative. Indeed, as one progresses along the spectrum from participants with the least irritability to those with the most irritability, the association between Δ*t*_0_ and amygdala-connectivity shifts from a positive to a negative correlation. Thus, while on average participants showed greater Δ*t*_0_ (i.e., indicates being slower in the incongruent vs. the congruent condition), youth with relatively high irritability demonstrated decreased engagement of regulatory circuitry for the incongruent vs. congruent contrast, while youth with relatively low irritability demonstrated increased engagement of neural regulatory circuitry.

Our findings are consistent with prior connectivity-based studies showing that amygdala connectivity to frontal regions, specifically the IFG, is associated with regulatory processing of emotional cues [25, 26, 58–60]. Here, youth who were relatively high on both the irritability spectrum and Δ*t*_0_ showed a relatively weak coupling between neural regions implicated in effective emotion regulation [61]. Non-irritable youth did not show this differential neural engagement. One potential interpretation is that decreased neural regulation in the context of aberrant attentional processing is maladaptive and may be a risk factor for irritability. It is also possible that reduced top-down engagement develops as a consequence of prolonged irritability, suggesting that experiencing clinical symptoms may have downstream neural effects. Both interpretations speak to aberrant neural regulation as characteristic of irritability uncovered by the current study. Future studies aiming to replicate these findings may include a longitudinal component to help elucidate a potential causal association.

We observed similar patterns of connectivity between the amygdala and subcortical regions implicated in the integration of emotional and attentional processing [62–64]. The pulvinar has been previously linked to fast, automatic visual recognition of aversive stimuli, both in non-human primates and in humans [65–67]. The insula has been previously identified as a region associated with threat processing and bottom-up detection of salient events [68]. Amygdala connectivity with the insula is assumed to allow the engagement of the salience network to deploy attention when threats appear [69, 70].

The consistency of the current findings across various brain regions implicated with emotional and attentional processing highlights robustness in identifying a potential global neural regulatory deficit in a sub-group of irritable youth. Our findings are aligned with previous phenotypic, behavioral, and neural observations. Clinically, irritable youth have a low threshold for reactive aggression, and demonstrate approach responses to emotionally-relevant stimuli [71]. Behaviorally, youth with irritability or elevated trait anger perceive neutral and ambiguous faces as more threatening and angry [7, 9, 72]. Neurobiologically, when processing angry faces, youth with irritability demonstrate aberrant amygdala, prefrontal, and executive attention network activation [6, 11].

Though the current study did not identify a direct link between irritability and attention bias, we found this association to be qualified by brain connectivity, reflected by the presence of a three-way interaction. Additionally, previous studies showed cognitive biases towards anger-related cues in youth with irritability and elevated trait anger [7–10]. Accumulating data on the neural bases of attentional processing associated with angry cues and its relation to irritability could potentially contribute targets for intervention in the future; however, more research and replications are needed. There is a significant literature demonstrating the efficacy of attention bias modification training (ABMT) using the dot-probe task in decreasing attention bias in anxiety [73]. This training aims to teach individuals to shift attention away from task-irrelevant negative cues. For example, White et al. [27] showed that ABMT was most effective in anxious patients with elevated attentional bias who also presented abnormal amygdala-insula connectivity. Interestingly, we found an analog association among Δ*t*_0_, decreased amygdala-insula connectivity, and irritability. Therefore, future work might examine whether irritable youth with disrupted amygdala-insula/IFG connectivity may benefit from a similar intervention. However, additional work is first needed to further explore the behavior and neural underpinnings of attention bias in irritability.

This study has several strengths. First, our task engaged relevant attentional and regulatory brain regions. The brain activation finding, showing association between greater Δ*t*_0_ and decreased activation in the right precuneus, is consistent with previous studies demonstrating the role of the precuneus during attentional shifting to emotional content [74], and with visual attention and inhibitory control [75–77]. Second, our data includes a large sample of well-characterized youth presenting a wide variation in irritability. Third, the current results in combination with those of Price and colleagues [22] validate the *t*_0_ parameter as a measure of attentional orienting with a standard DDM and support its added value in characterizing aberrant attention processing in pediatric psychopathology. This study provides converging evidence that cue-related processes occurring before probe onset are implicated in pediatric affective psychopathology. It has important theoretical implications for interpreting the dot-probe task and demonstrates the utility of computational modeling for interpreting cognitive tasks. Fifth, integrating computational strategies may offer a more precise methodological approach to capture anger-related attentional processing in a well-studied task, and its associations with clinical symptoms. The few findings we observed with the traditional attention bias scores based on raw response time (eResults 3) were distinct from those found with Δ*t*_0_. DDM can be used in future studies to quantify anger-related attention bias and assess changes in this parameter following interventions.

This study also has limitations. First, we found no direct association between irritability and DDM-derived attention bias at the behavioral level. While this is the first study examining Δ*t*_0_ in irritability, the non-significant association between these variables is inconsistent with previous data [7–10]. However, our three-way interaction reveals a more nuanced relationship between attention bias, irritability, and amygdala functional connectivity, indicating that neural abnormalities vary as a function of irritability and Δ*t*_0_. Discrepancies might also be associated with differences in task sensitivity across different levels of analyses, consistent with recent reports indicating that the subtraction score typically used to measure attention bias in the dot-probe task has poor psychometrics [78, 79]. By using a DDM approach to generate a latent parameter of attention bias, we overcome the limitations associated with relying on a difference value-based measure. More studies are needed to establish the reliability of Δ*t*_0_ and its potential superiority. Relative to the behavioral level, research demonstrates that neural connectivity during the task yields a more sensitive output [27]. In line with this, in the present study, task-based measures showed consistent associations with amygdala connectivity. Future studies aiming to replicate the current findings may enhance current knowledge regarding the sensitivity of the task across different levels of analyses.

Second, in a standard DDM, the *t*_0_ component represents both the attentional orienting process of interest and other processes including response preparation and motor execution. By contrasting congruent vs. incongruent conditions, we were able to isolate these processes related to the condition differences; however, we cannot precisely disentangle the different extra-decisional processes that may have influenced the findings, if indeed these are affected by congruency. Additionally, findings should prompt further modeling of the attentional effects on decision processing itself [23]. A question remains whether attentional shifts are reflected solely in *t*_0_, or also in the drift rate during evidence accumulation. To best identify the effects of attentional dynamics across the whole dot-probe trial, a revision to the standard DDM is likely required. This avenue could be further explored in future studies using other diffusion models [80].

Third, participants differed in psychotropic medication exposure. Although this speaks to ecological validity, it might confound our results, as medications may have differential effects on young brains and we could not directly disentangle the effects of medication usage from psychopathology. However, most participants (74.18%) were medication free. Importantly, our post-hoc analyses including medication status as a covariate indicated that all reported findings remained significant. We also replicated our behavioral and fMRI analyses limited to the large unmedicated sub-set of participants (see eResults 4). All original reported patterns remained within this sample.

A fourth limitation in the current study relates to the generalizability of the findings. Nearly a quarter of the acquired sample were excluded from analyses for a variety of reasons specified in the Supplement, including poor quality of the data or a failure to converge for the DDM. Although considered appropriate exclusion criteria, and consistent with previous literature [6, 27, 81], these may affect the generalizability of the findings. An additional consideration is the age range and the puberty status of the current sample. Little is known about the effects of pubertal status on neural systems of threat/anger processing. Some prior research suggests that pubertal maturation is associated with aberrant neural activity in brain regions implicated in emotional processing [82, 83]. In the current data, we adjusted for age, as well as for pubertal status, with the original findings surviving these inclusions (see eResults 5). The current sample consisted of mainly young adolescents in the early- to mid- stages of puberty (see Table 1). Longitudinal studies are needed to elucidate the effect of pubertal maturation on neural emotional processing, and our findings cannot be generalized to the entire span of puberty.

Fifth, findings should be interpreted with caution considering recent work indicating that preprocessing and modeling choices can meaningfully influence results in neuroimaging analyses in general, and gPPI specifically [84, 85]. In the current study, all specifications were determined a-priori, and established and off-the-shelf procedures were selected, reflecting the standard in both neuroimaging and DDM [6, 27, 49, 54]. These factors potentially reduce sensitivity to discrepancies [86]. The relatively large sample size in the present study may also mitigate some sensitivity concerns. Future studies applying multiverse approaches [87] and independent replications are needed to further evaluate the robustness of the results. Similarly, while our focus on amygdala connectivity was hypothesis-driven, seed-based connectivity approaches have limitations. Other approaches examining connectivity of large-scale networks across the brain may serve as a next step in providing an additional explanation of individual differences in irritability [88]. For example, Scheinost and colleagues [88] used a connectome-based predictive modeling to identify predictive networks of irritability. Whereas the present study explored the role of amygdala circuitry in attentional processing, future studies applying data-driven whole-brain networks analyses are needed to identify which additional brain regions play a role for aberrant processing as it relates to irritability.

In sum, this is the first study that applies DDM on an established attention task during concurrent fMRI recording to examine the neurobiology of anger-related attentional processing in a transdiagnostic sample of youth with varying levels of irritability. The current findings reflect an interplay between irritability and the way in which attentional processing of angry faces is linked to brain circuits, suggesting that youth with high irritability present with a dysfunction in the way that those circuits function when attention deployment is biased towards angry face stimuli. Future studies could further determine the extent to which this aberrant neuro-behavioral coupling underlies reactive aggression in irritability, test the efficacy of attentional training on irritability, and examine changes in amygdala connectivity to regions implicated in emotional and attentional regulation over the course of effective treatment and symptom remission.

## Supplementary information

Supplementary Materials
