# Supplementary material for: Context-dependent amygdala–prefrontal connectivity during the dot-probe task varies by irritability and attention bias to angry faces
Source: Neuropsychopharmacology. 2022 Jun 1;47(13):2283–91. doi: 10.1038/s41386-022-01307-3 (PMC9630440; doi:10.1038/s41386-022-01307-3)
Supplement: Supplementary file 1 — Supplementary Materials [file 41386_2022_1307_MOESM1_ESM.docx]

Supplementary Materials

**eMethods 1.** Eligibility and exclusion criteria, Dot-probe specifications, DDM specifications

**eMethods 2.** Affective Reactivity Index (ARI) and Screen for Child Anxiety Related Emotional Disorders (SCARED) questionnaires

**eTable 1.** Descriptive statistics of DDM parameters

**eFigure 1:** Model fit examination based on Quantile-Probability Plot

**eFigure 2:** Quantile-Probability Plot of empirical values of a single participant in whom the model failed to converge

**eMethods 3.** Neuroimaging acquisition, preprocessing and first-level analysis

**eFigure 3.** Whole-brain BOLD activity indicating negative association between Δt_0_ scores and activation in the right precuneus

**eResults 1.** Adding SCARED by ARI interaction term to the model

**eResults 2.** Post Hoc associations of neural activity and connectivity with irritability and Δt_0_, covarying ADHD Symptoms

**eTable 2.** Brain regions showing significant ARI by Δt_0_ interaction effects covarying ADHD Symptoms

**eResults 3.** Behavioral and fMRI analyses with the traditional attention-bias score

**eTable 3.** Brain regions showing significant effects for the traditional attention-bias score

**eResults 4**. Behavioral and fMRI analyses in the unmedicated sub-set of participants

**eResults 5**. Post Hoc associations of neural connectivity with irritability and Δt_0_, covarying for puberty status

**eReferences**

**eMethods 1**

**Eligibility and exclusion criteria**

Participants must be fluent in English and have an IQ above 70, as assessed by the Wechsler Abbreviated Scale of Intelligence (WASI) [1]. Participants were excluded if they were diagnosed with neurological disorder or unstable medical illness symptoms. Additionally, participants who meet DSM-5 criteria, as assessed by the Schedule for Affective Disorders and Schizophrenia for School-Age Children-Present and Lifetime Version (K-SADS-PL) [2], for schizophrenia, autism spectrum disorder, major depressive episode, posttraumatic stress disorder, and substance use within the preceding three months were also excluded. Participants were not eligible if they exhibit active suicidality or any cardinal bipolar symptoms, such as elevated or expansive mood, grandiosity or inflated self-esteem, and\or decreased need for sleep [3]. 113 participants were excluded out of the 464 enrolled to the study, due to technical issues during scanning (N=6), clinical findings upon scanning/poor quality of anatomical data (N=4), excessive in-scanner motion (N=25), poor task performance (accuracy<75%, N=38), or aborting the task (N=11). Siblings were randomly excluded as well (N=14). An additional N=15 participants were excluded from the analysis due to the model failing to converge in fastdm. This failure of convergence was due to an insufficient number of valid trials across task conditions or the lack of errors. Thus, simulations based on the extracted parameter estimates, could not be generated. Convergence tolerance parameters were at their default values [4]. See eFigure 2 for an example of QP values of empirical values of one participant in whom the model failed to converge. Comparing participants with an adequate model fit versus a model which failed to converge showed no differences between sub-groups in irritability levels, age, and sex distribution (*p*s >.093). Collectively, analyzed and unanalyzed groups did not differ in levels of irritability, age and sex distribution (*p*s >.648).

**Dot-probe specifications**

Each trial began with a fixation cross (500 milliseconds). Next, a pair of faces of an identical actor (angry-neutral or neutral-neutral, 500 milliseconds) was presented, followed by a probe (< or >, 1000 milliseconds) positioned in place of either the emotional or the neutral face (Figure 1). Participants were instructed to respond quickly and accurately by pressing the button that corresponded to the probe's direction. The angry face location and probe characteristics were counterbalanced. Trials were administered randomly in 2 runs, with a total of 80 congruent, 80 incongruent, and 80 neutral trials. Eighty fixation-only trials provided an additional baseline.

**Drift-diffusion model (DDM) specifications**

The DDM assumes decision making is driven by an evidence-accumulation process that begins at probe onset and evolves according to a Wiener drift-diffusion process. The decision process ends once the evidence reaches either an upper threshold $a$, which results in a correct response, or a lower threshold at zero, which results in an incorrect response. The response time is the time for the evidence to reach threshold, plus an extra-decisional time. The extra-decisional time on each trial is drawn from a uniform distribution with mean $t_{0}$ and range $s_{t}$. The drift rate on each trial is drawn from a normal distribution with mean $v$ and standard deviation $s_{v}$. The initial value of the evidence on each trial is drawn from a uniform distribution with mean $\left( z_{r} \right)$½ and range $a\cdot s_{zr}$. To make the other model parameters identifiable, the diffusion rate is traditionally fixed at $\sigma=0.1$.

In the current study, participants whose accuracy on the dot probe task fell below 75% were excluded (N=38). Omissions and incorrect trials, trials with extreme RTs (<150 or >2000 ms) or with RTs exceeding 2.5 SDs from the participant’s mean RT for the task were excluded; 8% of trials were discarded as a result [5-7]. DDM was then applied. Responses were coded as correct vs. incorrect [8]. Distributions of response times for correct and incorrect responses were compiled for each individual, for each of the three trial conditions (i.e., congruent, incongruent, neutral).$t_{0}$, the parameter of interest, was allowed to vary across task conditions (i.e., incongruent, congruent, and neutral). All other parameters were fixed across conditions. The response bias parameter, $z_{r}$, was fixed at ½ (no bias), in line with the correct–incorrect response coding. Maximum Likelihood (ML) was used as the estimation procedure [9].

All task conditions were included in the model (incongruent, congruent, and neutral). We adopted Voss and colleagues’ recommendation [10] and set differences in speed of response execution (*d*), and percentage of contaminants (*p*) to 0. All other model parameters other than $t_{0}$ ($v$, $a$, $s_{t}$, $s_{v}$, $s_{zr}$) were measured at the individual level, fixed across conditions within subject (see eTable 1). We run the following command using *fast-dm* tool:

method ml

precision 3

set d 0

set p 0

set zr 0.5

depends t0 stimulus

format stimulus RESPONSE TIME

To assess whether the model adequately fit the empirical data, we used the construct-samples tool of fast-dm to generate simulated datasets based on each participant’s parameter estimates, with 1000 iterations per condition. Quantile–probability plots were generated using MATLAB to examine quantiles in simulated and empirical data (see eFigure 1) [11]. Model fit showed an adequate account of response probability and RT via visual inspection.

**eMethods 2**

**Affective Reactivity Index (ARI) and Screen for Child Anxiety Related Emotional Disorders (SCARED) questionnaires**

Irritability was assessed using the Affective Reactivity Index (ARI; 6-month version) parent-report and youth-report questionnaires, a reliable seven-item measure (possible range for first six items computed in total score is 0–12). Cronbach’s alpha coefficients for both youth- and parent-reported ARI in the current study were high (α=.89 and .91, respectively). Levels of anxiety were assessed using the parent- and youth-report questionnaires, a 41-item screening tool. SCARED has a possible range of 0–82 and has shown excellent internal consistency. Cronbach’s alpha coefficients for both youth- and parent-reported SCARED in the current study were high (both αs = .95). These clinical assessments were conducted within 3 months of the scan date, except for seven healthy controls and one participant with a diagnosis of ADHD, whose data were collected within 6 months.

**eTable 1. Descriptive Statistics of DDM Parameters**

| **Model Parameters** |  |  |  |  |
| --- | --- | --- | --- | --- |
| Varied across conditions and participants | | Incongruent | Congruent | Neutral |
|  |  | Mean (SD) | | |
| Extra-decisional time (*t_0_*) |  | .499 (.100) | .498 (.102) | .499 (.101) |
| Measured at the individual level Mean (SD) | |  |  |  |
| Drift rate ($v$) | 5.159 (2.505) |  |  |  |
| Decisional threshold separation (*a*) | 1.409 (.975) |  |  |  |
| Intertrial variability in extra-decisional time (*s_t_*) | .195 (.088) |  |  |  |
| Intertrial variability in drift rate ($s_{v}$) | .200 (.376) |  |  |  |
| Intertrial variability in response bias (*s_zr_*) | .571 (.288) |  |  |  |

**eFigure 1**

**Model Fit Examination Based on Quantile-Probability Plots**


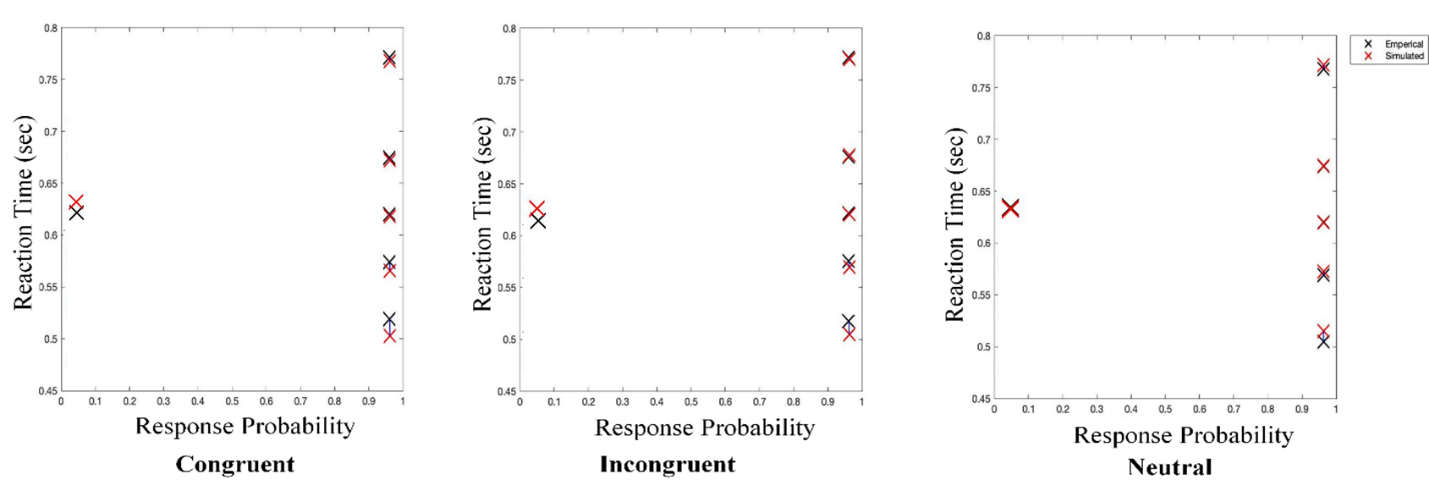


Xs indicate 10th, 30th, 50th, 70th, 90th percentiles of RT distributions. Black Xs represent empirical data and red Xs represent simulated data. In each plot, correct responses are shown at right (high probability) and errors are shown at left (low probability). Median scores were used for all errors due to low occurrence.

**eFigure 2**

**Quantile-Probability Plot of Empirical Values of a Single Participant in Whom the Model Failed to Converge**

**
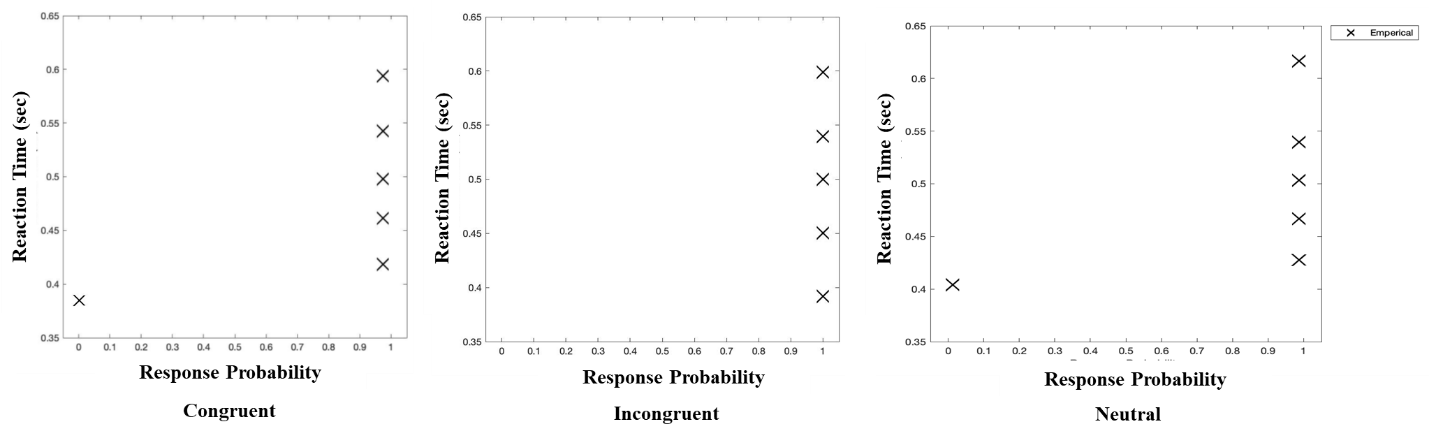
**

Xs indicate 10th, 30th, 50th, 70th, 90th percentiles of RT distributions. Black Xs represent empirical data. No simulated data is presented as CDF could not be generated for participants with data that failed to converge. In each plot, correct responses are shown at right (high probability) and errors are shown at left (low probability). Median scores were used for all errors due to low occurrence. This participant had no errors in the incongruent condition.

**eMethods 3**

**Neuroimaging acquisition, preprocessing and first-level analysis**

Blood oxygen level-dependent signal (BOLD) was measured using a T2-weighted gradient-echo pulse sequence (TR=2300 ms, TE=25 ms, flip angle=50°, field of view (FOV)=240 mm^2^, matrix=96×96, in-plane resolution of 2.5 × 2.5 mm, and either 41 or 42 contiguous 3-mm interleaved axial slices). Total acquisition time was 14 minutes. For co-registration and normalization, a high resolution, 3-dimensional MPRAGE spin-echo sequence was required. Images were collected with echo time/inversion time of minimum full echo time/725 ms, FOV of 220 mm, 256×192–pixel matrix, and bandwidth of 31.25 Hz per 256 voxels.

To allow the magnet to reach a steady state, the first four TRs of each run were discarded for a total of 180 TRs per run. The AFNI tool @SSwarper was used for skull stripping and for calculating the warp to the MNI152_2009 space template. Further standard pre-processing included despiking, slice-timing correction, distortion correction, normalization, and non-linear registration of echoplanar data to anatomical scans. Data were smoothed (kernel = 6.5 full-width-half-maximum), resampled to 2.5 mm isotropic voxels, and intensity scaled. For motion correction, volumes that had more than 10% outliers and TR pairs with a Euclidean norm motion derivative greater than 1 mm were censored prior to individual-level analyses. To be included in the analysis, no more than 20% of TRs across conditions could be censored, and average in-scanner motion could not exceed 0.25 mm after censoring [5,6].

First-level analyses were conducted by creating a GLM for each participant using procedures consistent with previous studies [5]. Brain activation across each task trial, with face stimuli signaling the beginning of each event, was estimated by deconvolving with AFNI's GAM basis function. Trials with incorrect responses and trials in which RTs were excluded in the behavioral processing were modeled as regressors of no interest. The six motion parameters were included as additional regressors. Left and right amygdala regions of interest (ROIs) were anatomically defined using the Harvard Oxford Atlas of the FMRIB Software Library (FSL) [12] and thresholded at 50% probability [13]. Only those voxels for which data existed for at least 90% of participants were included in the ROIs.

**eFigure 3**

**Whole-brain BOLD**

**
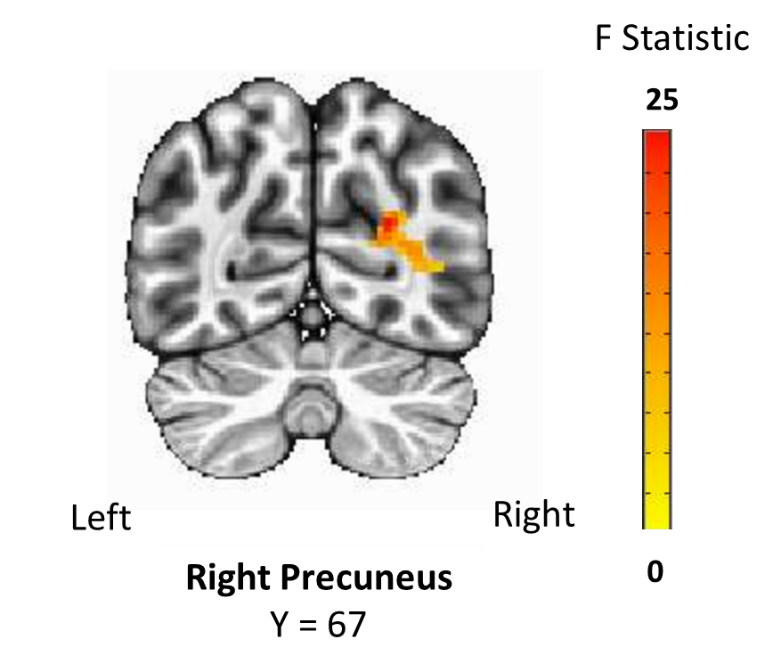
**

Whole-brain analysis revealed that increased Δt_0_ scores were negatively associated with activation in the right precuneus (β_350_=−.71, *t*_350_=−4.61, *p*<.001) during orienting to angry face stimuli. *k*=247 voxels; *p*<.005 whole-brain corrected.

**eResults 1**

**Adding ARI by SCARED interaction term to the model**

Supplementary group level analyses were conducted with adding SCARED by ARI interaction scores as an additional term to the model. Specifically, model included $\Delta t_{0}$, irritability score, anxiety score, interaction between SCARED and ARI, and interaction between each irritability/anxiety with$\Delta t_{0}$. The three-way interaction of ARI by SCARED by $\Delta t_{0}$ was also in the model. Similarly to the initial model, age, motion, sex, and head-coil channel during acquisition were entered to the model as covariates.

No additional findings to the initial model emerged. Specifically, no anxiety related findings emerged. The whole brain activation finding with $\Delta t_{0}$and four out of the six amygdala seed- based connectivity findings for ARI by $\Delta t_{0}$ interaction remained significant. Specifically, left amygdala connectivity with right insula and right IFG, and right amygdala connectivity with right caudate and right thalamus/pulvinar, survived this addition (*βs*_350_<−1.71, *ts*_350_<−4.86, *ps*<.001).

**eResults 2**

**Post hoc associations of neural activity and connectivity with irritability and** Δt_0_**, covarying ADHD Symptoms (N=312)**

Since research linking DDM to ADHD [14,15] and due to associations between irritability and ADHD [16,17], also in the current study (*r*_312_=0.38, *p*<.001), supplementary post hoc analyses were conducted controlling for levels of ADHD symptoms. 312 participants had completed additional ADHD measure using the Conners’ Parent Rating Scale [18]. Symptoms were assessed within 3 months of the imaging scan. Total scores on this measure were entered into the model. All findings remained significant (eTable 2).

**eTable 2. Brain regions showing significant interaction effects covarying ADHD Symptoms**

| Anger Incongruent vs. Congruent | t Statistic | P Value | Location |
| --- | --- | --- | --- |
| **Right Amygdala Seed Functional Connectivity** | | | |
| Δt_0_ by Irritability PC mean | −5.37 | <.001 | Right Caudate |
|  | −5.15 | <.001 | Right Thalamus/pulvinar |
| **Left Amygdala Seed Functional Connectivity** | | | |
| Δt_0_ by Irritability PC mean | −5.74 | <.001 | Right (anterior) Insula |
|  | −5.02 | <.001 | Right IFG |
|  | −4.75 | <.001 | Left IFG |
|  | −4.74 | <.001 | Left (anterior) Insula |

Abbreviations: Δt_0_ = extra-decisional time bias; ADHD = attention-deficit hyperactivity disorder; Note: ADHD symptoms were assessed using total scores on the Conners’ Parent Rating Scale [18]; *p*-value represents significance value from post hoc multivariate linear regression on mean BOLD signal for extracted cluster; Location represents anatomical overlap of cluster with region.

**eResults 3**

**Behavioral and fMRI analyses with the traditional attention-bias score**

Traditional RT-based attention bias scores were calculated by subtracting congruent trial mean RTs from incongruent trial mean RTs. First, a correlation was computed between $\Delta t_{0}$ and traditional attention bias scores and was found to be significant (*r*_s351_=0.38, *p*<.001). A multivariate linear regression model was conducted including irritability, anxiety, age, and sex as predictors and traditional attention bias scores as the outcome variable. Adjusting for covariates, no significant findings emerged for the association between irritability and traditional attention bias (*p*=.625).

To parallel the primary fMRI analysis, similar models to the one used with $\Delta t_{0}$ were executed with the traditional behavioral attention-bias score for the contrast of attentional orienting to angry face stimuli (angry incongruent vs. angry congruent). AFNI's 3dMVM included attention-bias, irritability scores, interaction between attention-bias and irritability scores as between subject independent variables. Age, motion, and anxiety scores, as well as sex and head-coil, were entered to the model as covariates.

Attention bias scores were associated with increased functional connectivity between the left amygdala and the left medial frontal gyrus (*β*_350_=.005, *t*_350_=3.46, *p*=.001) during orienting to angry face stimuli. In addition, a significant irritability by attention bias interactions were found for the right amygdala connectivity with the dorsal striatum (*β*_350_=−.002, *t*_350_=−4.81, *p*<.001) and the right precentral/superior temporal (*β*_350_=−.003, *t*_350_=−4.66, *p*<.001). Increased attention bias was associated with poor coupling between those regions, in youth high in irritability, with compared to youth low in irritability.

|  |  | | Peak MNI Coordinates (Icent) | | | |  |  |  |  |
| --- | --- | --- | --- | --- | --- | --- | --- | --- | --- | --- |
| Anger Incongruent vs. Congruent | | K | | X | Y | Z | Size, mm^3^ | t Statistic | P Value | Location |
| **Left Amygdala Seed Functional Connectivity** | | | | | |  |  |  |  |  |
| Attention Bias | | 80 | | 45 | −3 | 48 | 1,250 | 3.46 | =.001 | Left Medial Frontal Gyrus |
| **Right Amygdala Seed Functional Connectivity** | | | | | | | | | | |
| Attention Bias by Irritability PC mean | | 110 | | 25 | −11 | −4 | 1,718 | −4.81 | <.001 | Dorsal Striatum |
|  |  | 76 | | −52 | 4 | 3 | 1,187 | −4.66 | <.001 | Right Precentral/Superior Temporal |

**eTable 3. Brain regions showing significant main and interaction effects for the traditional attention-bias score.**

Abbreviations: PC = parent child; Note: *p*-value represents significance value from post hoc multivariate linear regression on mean BOLD signal for extracted cluster; Location represents anatomical overlap of cluster with region.

**eResults 4**

**Behavioral and fMRI analyses in the unmedicated sub-set of participants**

Same statistical analyses were conducted, limited to the unmedicated sub-set of participants (N=250). All the original reported findings were replicated with this sample. Specifically, a significant interaction was observed between $\Delta t_{0}$and irritability for left amygdala functional connectivity with bilateral IFG and insula (right IFG: β_242_=−2.04, t_242_=−3.97, p<.001; left IFG: β_242_=−2.25, t_242_ =−4.37, p<.001; right insula: β_242_=−2.55, t_242_ =−5.41, p<.001; left insula: β_242_=−1.83, t_242_=−3.67, p<.001), and for right amygdala functional connectivity with right caudate and right thalamus/pulvinar (right caudate: β_242_=−2.29, t_242_=−5.29, p<.001; right thalamus/pulvinar: β_242_=−1.85, t_242_=−4.13, p<.001). We also repeated the original behavioral analyses in the unmedicated sub-set of 250 participants. These analyses did not yield more significant results regarding the behavioral effects (p=0.650).

**eResults 5**

**Post Hoc associations of neural connectivity with irritability and Δt_0_, covarying for puberty status**

Supplementary post-hoc analyses were conducted adding puberty status score to the model. 237 participants had completed the Tanner scale [19]. Scores are ranging from 1 to 5 (1=pre-pubertal, 5=pubertal development complete). Symptoms were assessed within 6 months of the imaging scan. Total scores on this measure were entered into the model. All original findings remained significant. Specifically, a significant interaction was observed between $\Delta t_{0}$and irritability for left amygdala functional connectivity with bilateral IFG and insula (right IFG: β_228_=−1.81, t_228_=−3.51, p=.001; left IFG: β_228_=−1.71, t_228_ =−3.20, p=.002; right insula: β_228_=−1.45, t_228_ =−3.31, p=.001; left insula: β_228_=−1.36, t_228_=−2.80, p=.002), and for right amygdala functional connectivity with right caudate and right thalamus/pulvinar (right caudate: β_228_=−1.81, t_228_=−4.13, p<.001; right thalamus/pulvinar: β_228_=−1.91, t_228_=−4.17, p<.001).

**eReferences**

1 Wechsler D. Manual for the Wechsler abbreviated intelligence scale (WASI)*.* The Psychological Corporation: San Antonio, TX; 1999.

2 Kaufman J, Birmaher B, Brent D, Rao U, Flynn C, Moreci P, et al. Schedule for Affective Disorders and Schizophrenia for School-Age Children Present and Lifetime version (K-SADS-PL): Initial reliability and validity data. J Am Acad Child Psy. 1997;36(7):980-88.

3 Wiggins JL, Brotman MA, Adleman NE, Kim P, Oakes AH, Reynolds RC, et al. Neural Correlates of Irritability in Disruptive Mood Dysregulation and Bipolar Disorders. Am J Psychiatry. 2016;173(7):722-30.

4 Voss A, Voss J. Fast-dm: a free program for efficient diffusion model analysis. Behav Res Methods. 2007;39(4):767-75.

5 Kircanski K, White LK, Tseng WL, Wiggins JL, Frank HR, Sequeira S, et al. A Latent Variable Approach to Differentiating Neural Mechanisms of Irritability and Anxiety in Youth. JAMA Psychiatry. 2018;75(6):631-39.

6 White LK, Britton JC, Sequeira S, Ronkin EG, Chen G, Bar-Haim Y, et al. Behavioral and neural stability of attention bias to threat in healthy adolescents. Neuroimage. 2016;136:84-93.

7 Britton JC, Suway JG, Clementi MA, Fox NA, Pine DS, Bar-Haim Y. Neural changes with attention bias modification for anxiety: a randomized trial. Soc Cogn Affect Neurosci. 2015;10(7):913-20.

8 Price RB, Brown V, Siegle GJ. Computational Modeling Applied to the Dot-Probe Task Yields Improved Reliability and Mechanistic Insights. Biol Psychiatry. 2019;85(7):606-12.

9 Voss A, Nagler M, Lerche V. Diffusion models in experimental psychology: a practical introduction. Exp Psychol. 2013;60(6):385-402.

10 Voss A, Voss J, Lerche V. Assessing cognitive processes with diffusion model analyses: a tutorial based on fast-dm-30. Front Psychol. 2015;6:336.

11 Biscione V. Quantile Probability Plot. 2021. <https://www.mathworks.com/matlabcentral/fileexchange/49779-quantile-probability-plot>. Accessed April 8 2021 2021.

12 Jenkinson M, Beckmann CF, Behrens TE, Woolrich MW, Smith SM. Fsl. Neuroimage. 2012;62(2):782-90.

13 Amunts K, Kedo O, Kindler M, Pieperhoff P, Mohlberg H, Shah NJ, et al. Cytoarchitectonic mapping of the human amygdala, hippocampal region and entorhinal cortex: intersubject variability and probability maps. Anat Embryol (Berl). 2005;210(5-6):343-52.

14 Shapiro Z, Huang-Pollock C. A diffusion-model analysis of timing deficits among children with ADHD. Neuropsychology. 2019;33(6):883-92.

15 Fosco WD, White CN, Hawk LW, Jr. Acute Stimulant Treatment and Reinforcement Increase the Speed of Information Accumulation in Children with ADHD. J Abnorm Child Psychol. 2017;45(5):911-20.

16 Shaw P, Stringaris A, Nigg J, Leibenluft E. Emotion dysregulation in attention deficit hyperactivity disorder. Am J Psychiatry. 2014;171(3):276-93.

17 Cardinale EM, Freitag GF, Brotman MA, Pine DS, Leibenluft E, Kircanski K. Phasic Versus Tonic Irritability: Differential Associations With Attention-Deficit/Hyperactivity Disorder Symptoms. J Am Acad Child Adolesc Psychiatry. 2021.

18 Conners CK. Conners 3^rd^ edition manual. Multi-Health Systems, Inc., North Tonawanda, Canada, 2008.

19 Marshall WA, Tanner JM. Growth and Physiological Development during Adolescence. Annu Rev Med. 1968;19:283-&.
